# Supplementary material for: Tissue and liquid biopsy profiling reveal convergent tumor evolution and therapy evasion in breast cancer
Source: Nat Commun. 2022 Dec 5;13:7495. doi: 10.1038/s41467-022-35245-x (PMC9723105; doi:10.1038/s41467-022-35245-x)
Supplement: Supplementary file 1 — Supplementary Information [file 41467_2022_35245_MOESM1_ESM.pdf]

Tissue and liquid biopsy profiling reveal convergent tumor evolution and therapy evasion in breast cancer

SUPPLEMENTARY FIGURES

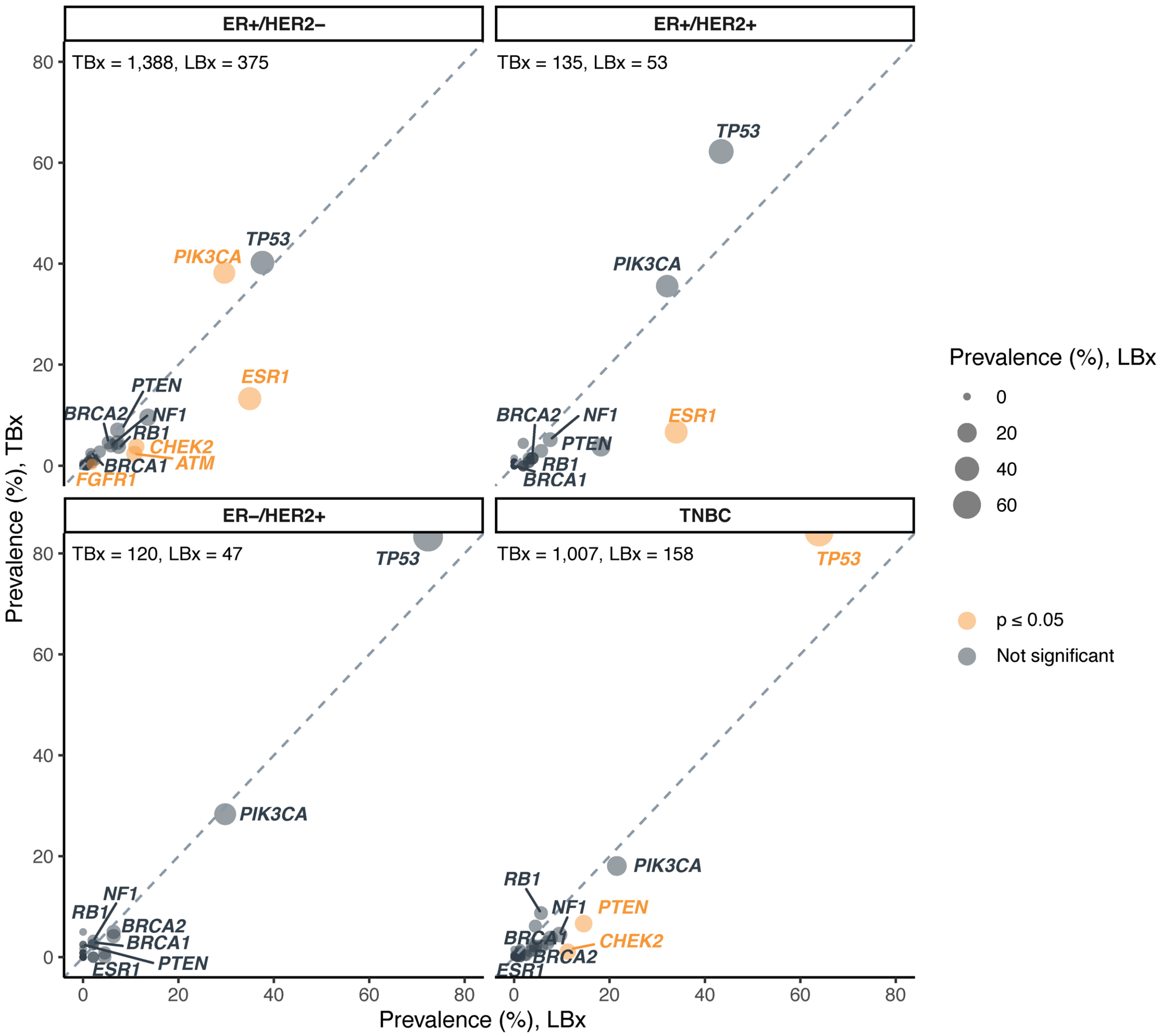

Supplementary Figure 1. Prevalence of point mutations and indels in tissue and liquid biopsy samples across different receptor subtypes. Prevalence of point mutations and indels in each platform (tissue biopsy, TBx; liquid biopsy, LBx) based on the receptor subtype: ER+/HER2-, ER+/HER2+, ER-/HER+ and TNBC. The total number of TBx and LBx cases evaluated in each receptor subtype are provided. The size of each data point is scaled to the gene alteration prevalence in LBx. Genes identified to have a statistically significant difference in prevalence between TBx and LBx are shown in orange. Additionally, commonly occurring gene alterations (*TP53*, *BRCA1*, *BRCA2*, *PIK3CA*, *ESR1*, *RB1*, *NF1*, *PTEN*) are labelled in each plot. The difference in alteration prevalence was determined by a two-sided Fisher's exact test, corrected for multiple hypothesis testing (FDR). Statistically significance was set at a FDR-corrected $p \leq 0.05$.

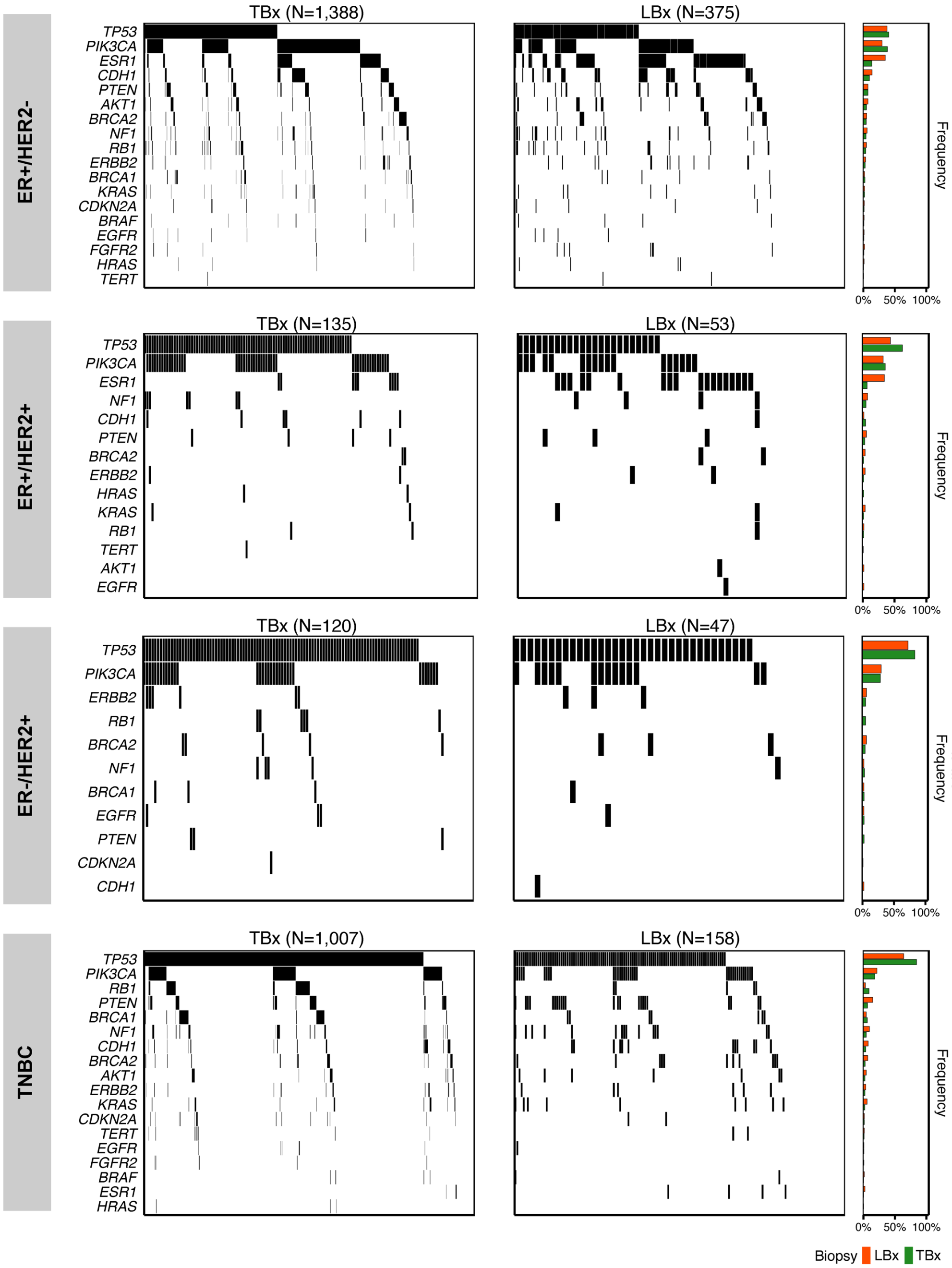

Supplementary Figure 2. Patterns of co-occurrence and mutual exclusivity between point mutations and indels detected in tissue and liquid biopsy samples across different receptor subtypes. Oncoplot displaying patterns of point mutations and indels in each platform (tissue biopsy, TBx; liquid biopsy, LBx) based on the receptor subtype: ER+/HER2-, ER+/HER2+, ER-/HER+ and TNBC. Commonly occurring gene alterations in the overall cohort are plotted (*TP53, PIK3CA, ESR1, CDH1, PTEN, NF1, BRCA2, AKT1, ERBB2, BRCA1, KRAS, CDKN2A, TERT, FGFR2, BRAF, EGFR, HRAS, RB1*). If a gene alteration was not detected in any sample within a receptor subtype, it was excluded from the plot. The total number of TBx and LBx cases evaluated in each receptor subtype are also provided. Alteration frequency for each of the assessed genes is presented as a bar plot on the right.

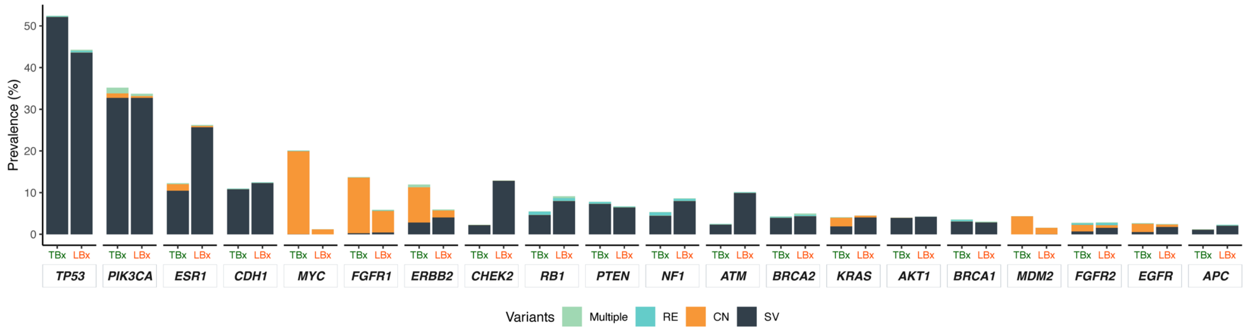

Supplementary Figure 3. Prevalence of gene alterations across all variant classes in tissue and liquid biopsy samples. Bar plot representing the prevalence of the twenty most frequently altered genes in each platform (tissue biopsy, TBx; liquid biopsy, LBx). Each bar is stacked based on all identified classes of variants within a gene: short variants (SV), including base substitutions and short indels, copy number alterations (CN) and rearrangements (RE). Samples exhibited more than one class of alteration are grouped into the 'Multiple' category. Sensitivity to capture copy number alterations and rearrangements is reduced in the LBx platform.

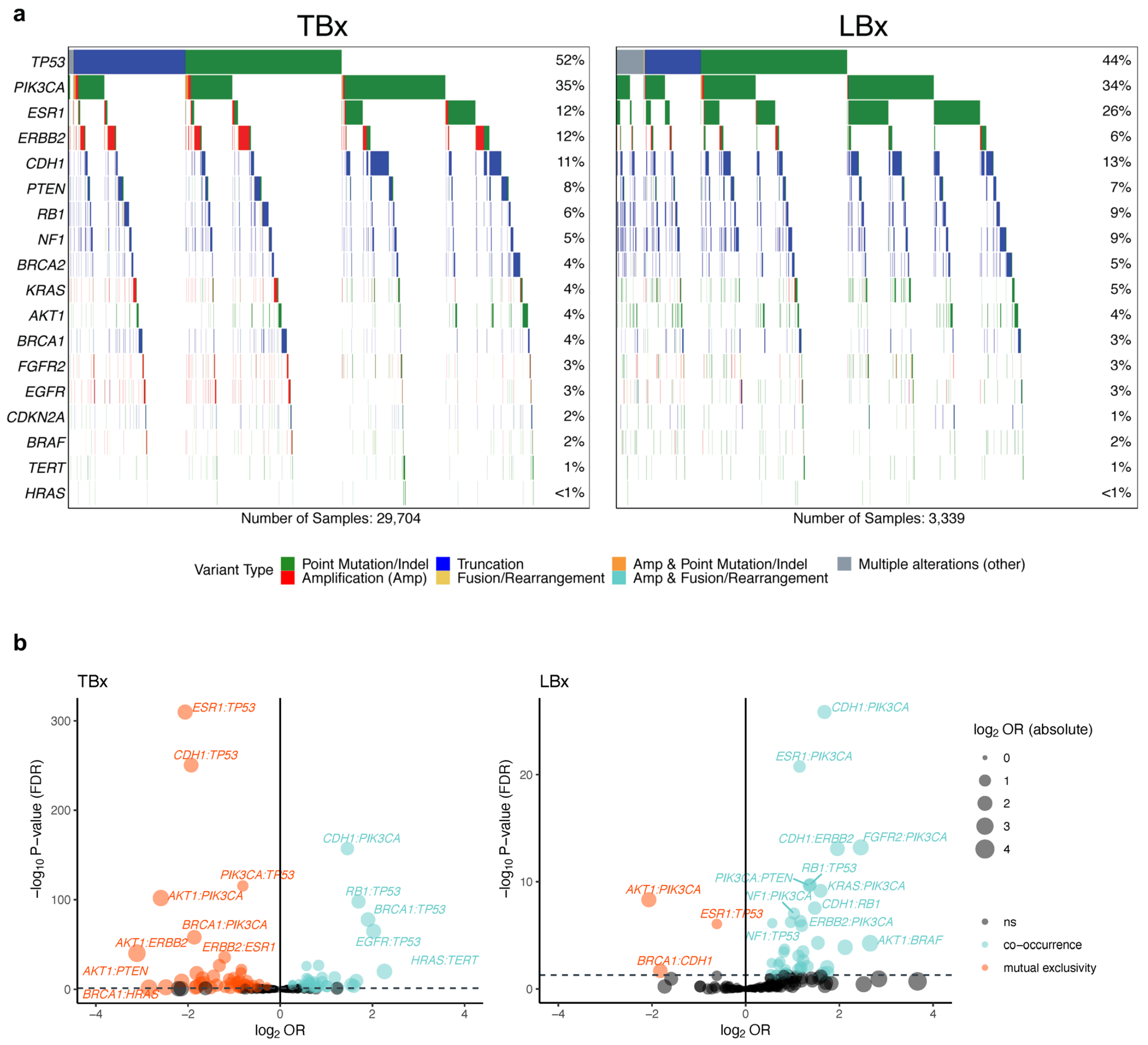

Supplementary Figure 4. Patterns of co-occurrence and mutual exclusivity between different gene alterations in tissue and liquid biopsy samples. (a) Oncoplot of the most frequently mutated genes in each platform – tissue biopsy, TBx (left) and liquid biopsy, LBx (right). Each patient is represented in this plot. The color denotes different detected variant types. The frequency of each gene alteration detected across the two platforms is also listed. **(b)** Volcano plot displaying the relationship between gene alterations in each platform – TBx (left) and LBx (right). A two-sided Fisher's exact test with FDR correction was applied for this analysis. Log 2 transformed odd's ratio (OR) and log 10 transformed P-value are shown for each pair of gene alterations assessed. The dashed line represents a FDR-corrected P-value of 0.05. Statistically significant patterns of co-occurrence (blue) and mutual exclusivity (orange) are displayed above the dashed horizontal line. Gene alteration pairs that were not statistically significant (ns) are shown in black. Select gene pairs are labelled to enhance viewability; additional information is available in Table S6 accompanying this analysis.

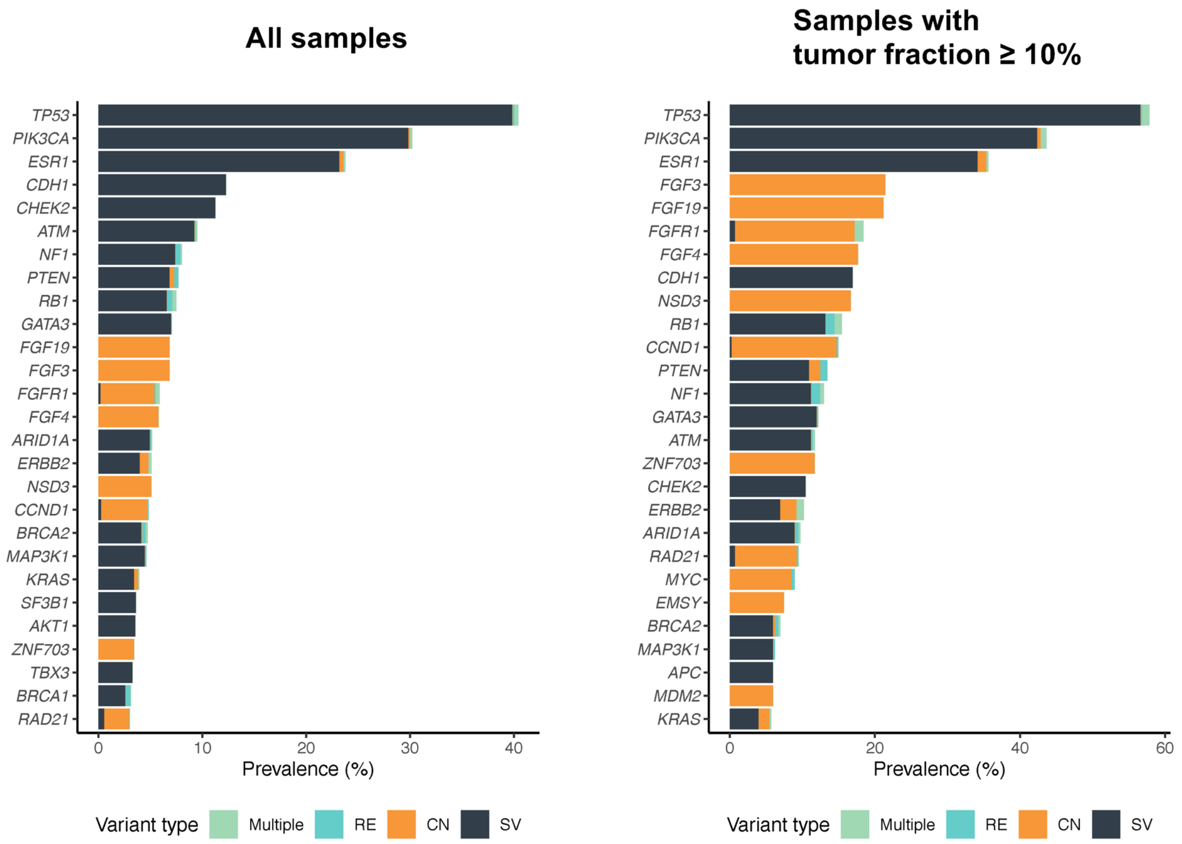

Supplementary Figure 5. Prevalence of gene alterations in liquid biopsies profiled using the FoundationOne®Liquid CDx assay. Prevalence of the gene alterations identified in the recently approved 324-gene panel FoundationOne®Liquid CDx profling assay[1] for all 1,430 available breast cancer samples (left) and a subset of 401 samples with an estimated tumor fraction of at least 10%, using the tumor fraction estimator approach (right) (*See Methods*). Each bar is stacked based on all identified classes of variants within a gene: short variants (SV), including single nucleotide variants and short indels, copy number alterations (CN) and rearrangements (RE). Samples exhibited more than one class of alteration are grouped into the 'Multiple' category. Alterations in *ASXL1*, *DNMT3A*, and *TET2* were excluded from this analysis, as likely inferred clonal hematopoiesis (iCH)-associated alterations[2].

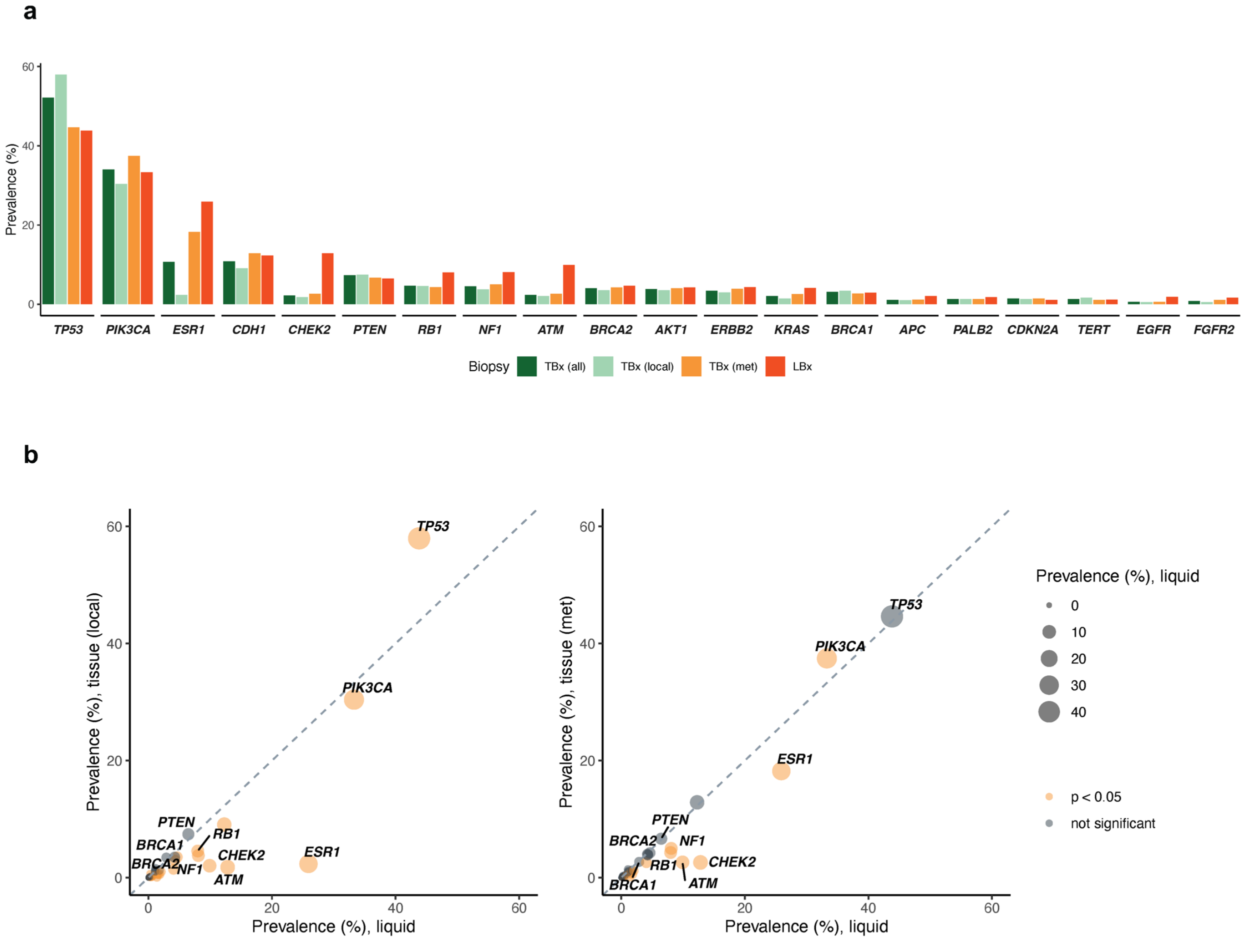

Supplementary Figure 6. Comparison of mutation profiles in liquid biopsy with local and metastatic tissue biopsy. (a) Prevalence of short variants for the twenty most frequently altered genes in each platform (tissue biopsy, TBx; liquid biopsy, LBx). In addition to all TBx samples (N = 29,704), we assessed subsets of local (N = 9,860) and metastatic (met; N = 13,811) TBx samples, where available. **(b)** Comparison of prevalence for all genes assessed in LBx (x-axis) and TBx (y-axis), examined separately for local (left) and met (right) TBx samples. Genes with statistically significant alteration prevalence between the two assays, based on a two-sided Fisher's exact test with FDR correction (p-value ≤ 0.05) have been shown in orange.

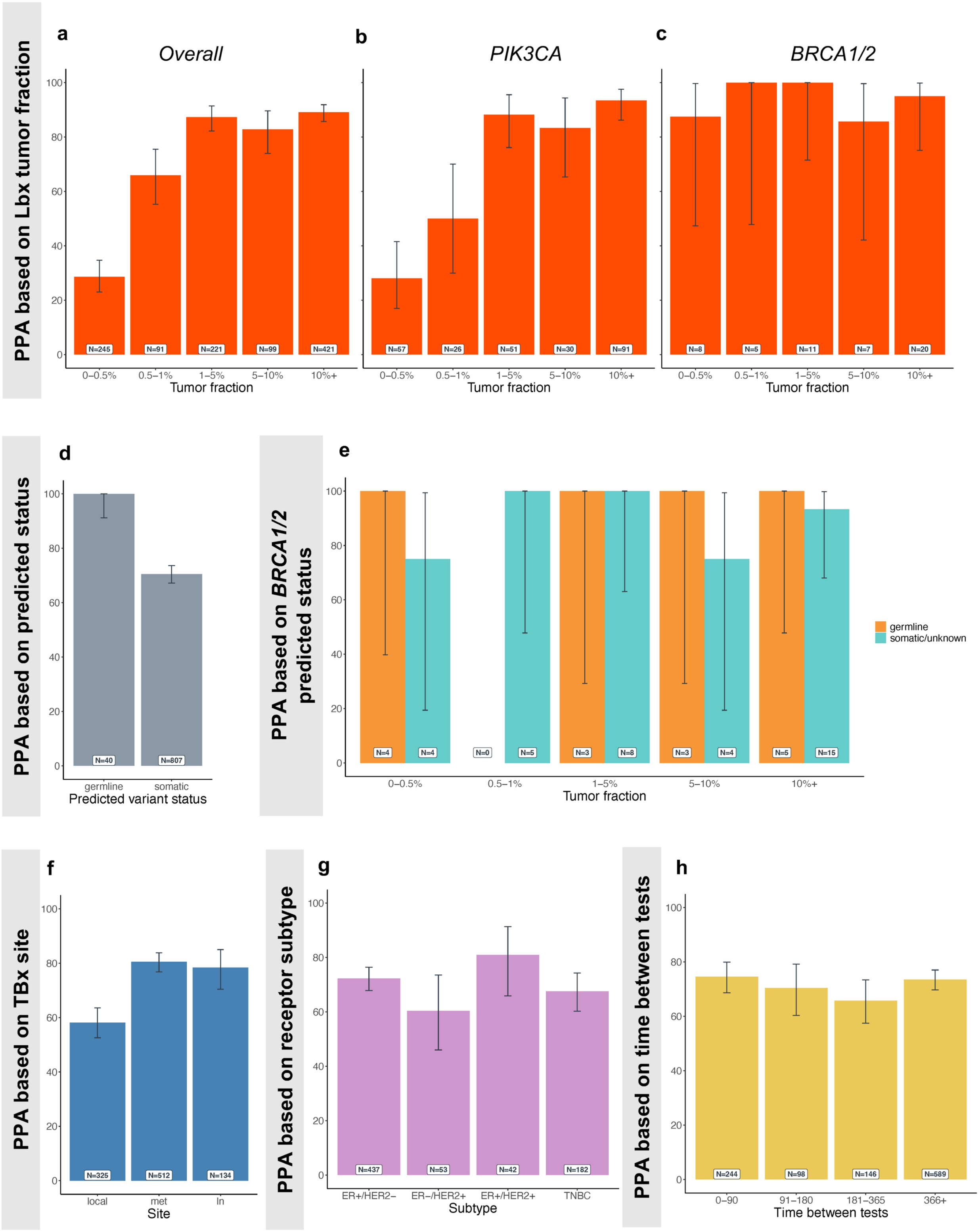

Supplementary Figure 7. Percent positive agreement in 712 patient-matched tissue biopsy (TBx) and liquid biopsy (LBx) samples. PPA of LBx, using TBx as a reference, stratified on **(a)** estimated tumor fraction in LBx for all short variants, **(b)** estimated tumor fraction in LBx for *PIK3CA* short variants, **(c)** estimated tumor fraction in LBx for *BRCA1/2* short

variants, **(d)** predicted germline/somatic status, using a consensus germline approach whereby recurrent alterations that are nearly always flagged as germline in our dataset were labeled as ‘germline’, **(e)** *BRCA1/2* variants separated by their germline/somatic status, **(f)** site of tissue biopsy, **(g)** receptor subtype, **(h)** time between tests. Error bars represent the 95% binomial confidence interval centered on the PPA; the number of variants detected in TBx for each bin are displayed.

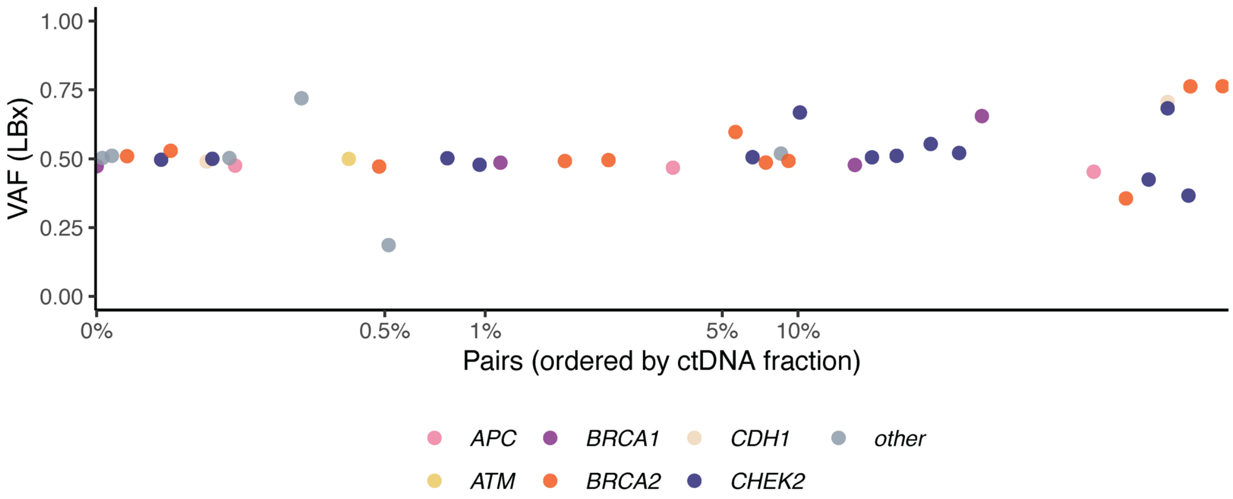

Supplementary Figure 8. Distribution of predicted germline alterations in patient-matched tissue and liquid biopsy samples. Presence and variant allele frequency for predicted germline alterations detected by liquid biopsy assays in patient-matched tissue and liquid biopsy pairs. A consensus germline approach whereby recurrent alterations that are nearly always flagged as germline in our dataset were labeled as ‘germline’. Patient-matched biopsy pairs (x axis) are ordered by their estimated tumor fraction in the liquid biopsy sample. Predicted germline alterations were detected, regardless of tumor fraction estimated in the liquid biopsy sample.

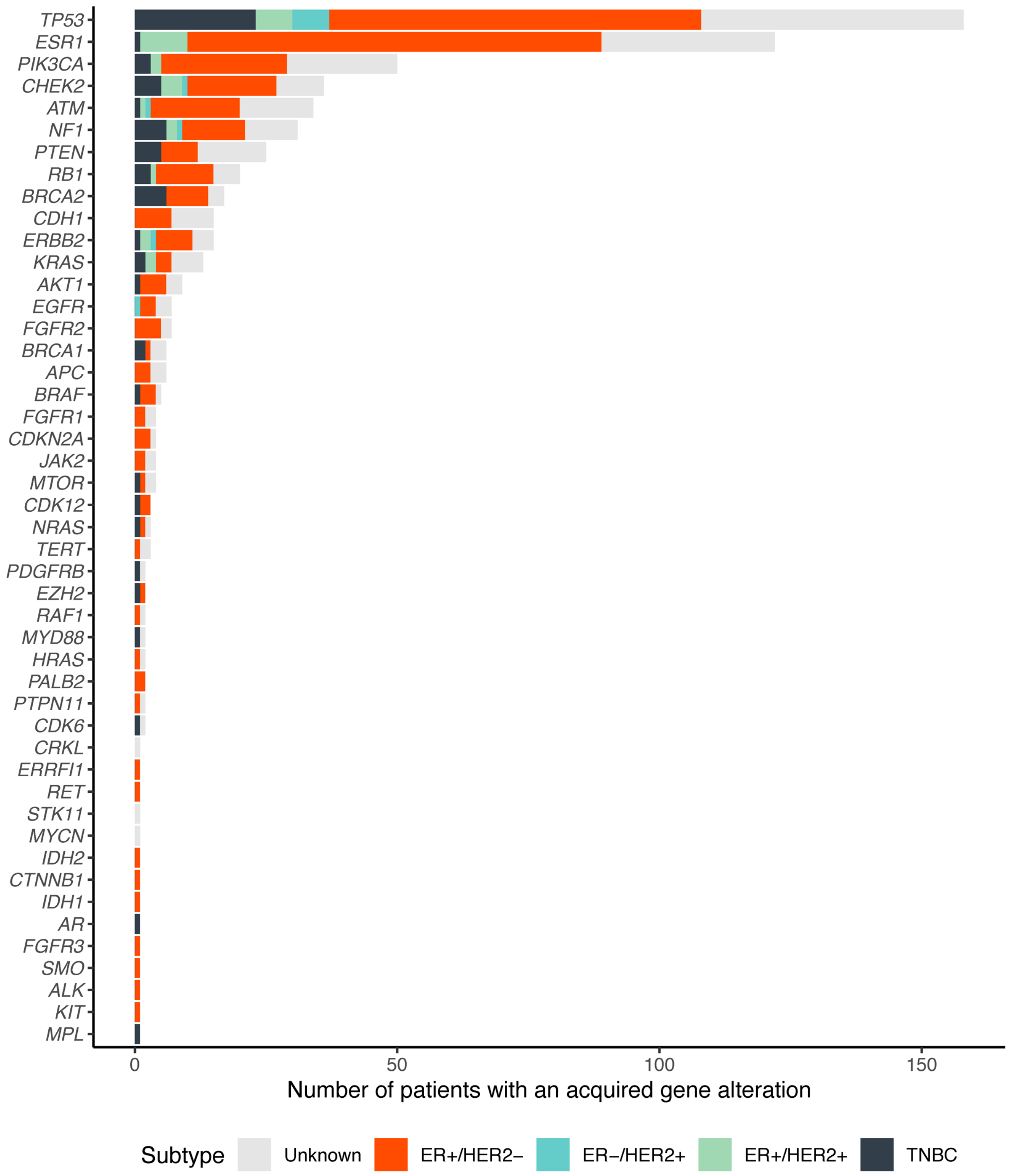

Supplementary Figure 9. Spectrum of liquid biopsy-specific acquired short variants based on receptor status. The spectrum of acquired short variants in liquid biopsies, following an initial tissue biopsy, in our patient-matched biopsy pair cohort. Shown here is the number of patients with each gene alteration based on the receptor subtype status in the baseline tissue biopsy sample – ER+/HER2+, ER+/HER2-, ER-/HER2+, TNBC (where available).

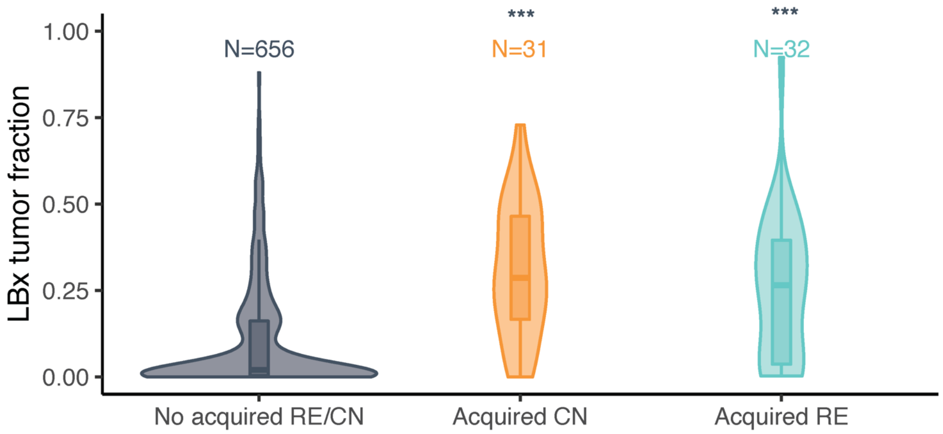

Supplementary Figure 10. Distribution of tumor fraction in liquid biopsies, based on the status of acquired copy number alterations and rearrangements. Box and violin plots displaying the distribution of the tumor fraction for the liquid biopsy (LBx) samples based on the detection of acquired copy number alterations (CN) and rearrangements (RE). LBx samples, where these complex events were detected, showed higher tumor fraction than samples lacking acquired CN/RE ($p=1.8x10^{-9}$ and $p=1.9x10^{-6}$ for those with acquired CN and RE respectively; *** : $p < 0.001$, two-sided Wilcoxon test). Each box plot displays the interquartile range (IQR), with the lower and upper boundaries representing the 25th and 75th percentile; the line within the box represents the median and the whiskers extend to ±1.5 x IQR.

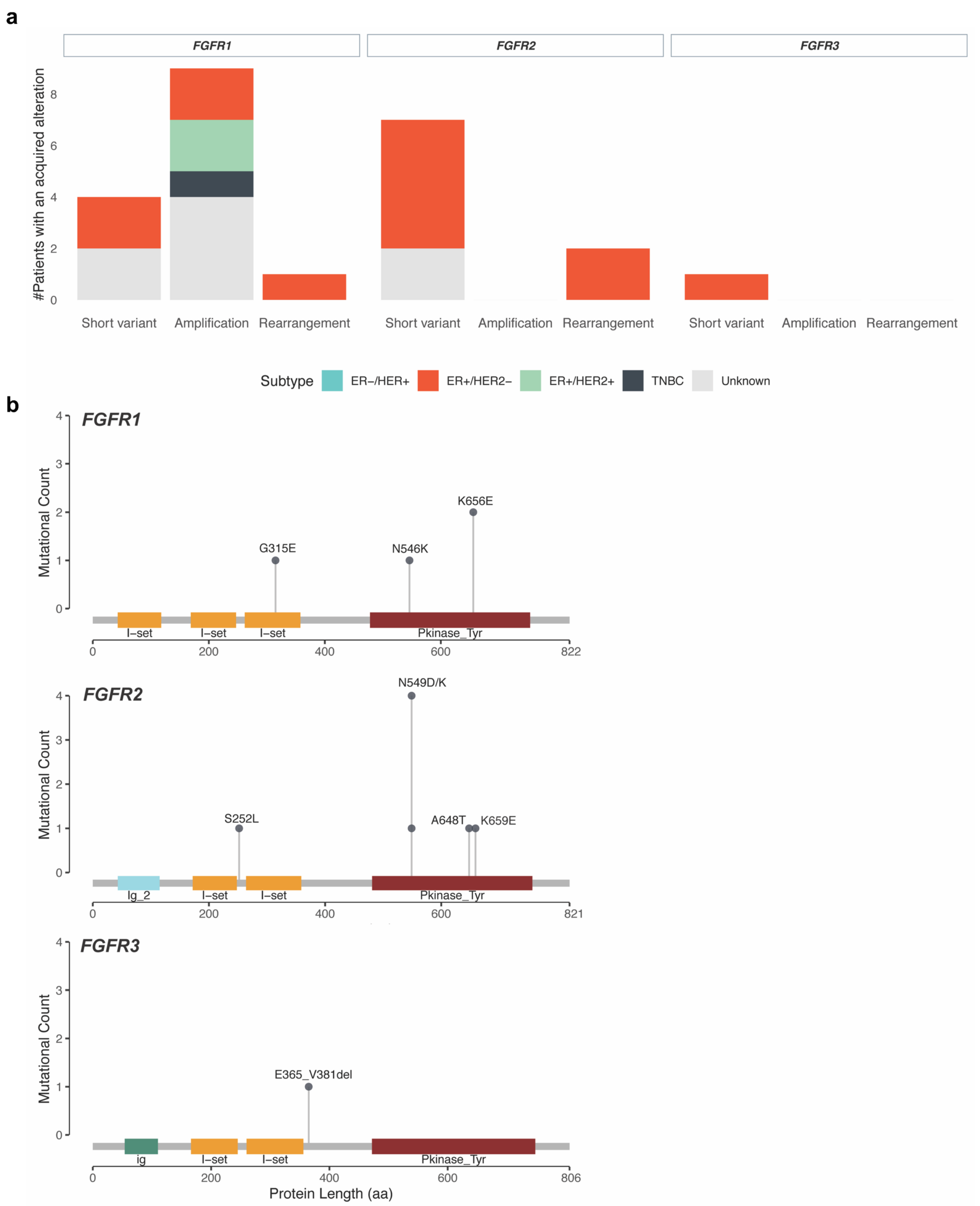

Supplementary Figure 11. Spectrum of liquid biopsy-specific acquired alterations in *FGFR1/2/3* genes. (a) All observed instances of acquired short variants, copy number alterations and rearrangements in *FGFR1*, *FGFR2* and *FGFR3* detected in liquid biopsies (LBx), following an initial tissue biopsy, in patient-matched biopsy pairs are shown. Receptor status is annotated, based on availability. **(b)** Lollipop plots displaying the acquired short variants within *FGFR1*, *FGFR2* and *FGFR3* detected unique to the follow-up LBx.

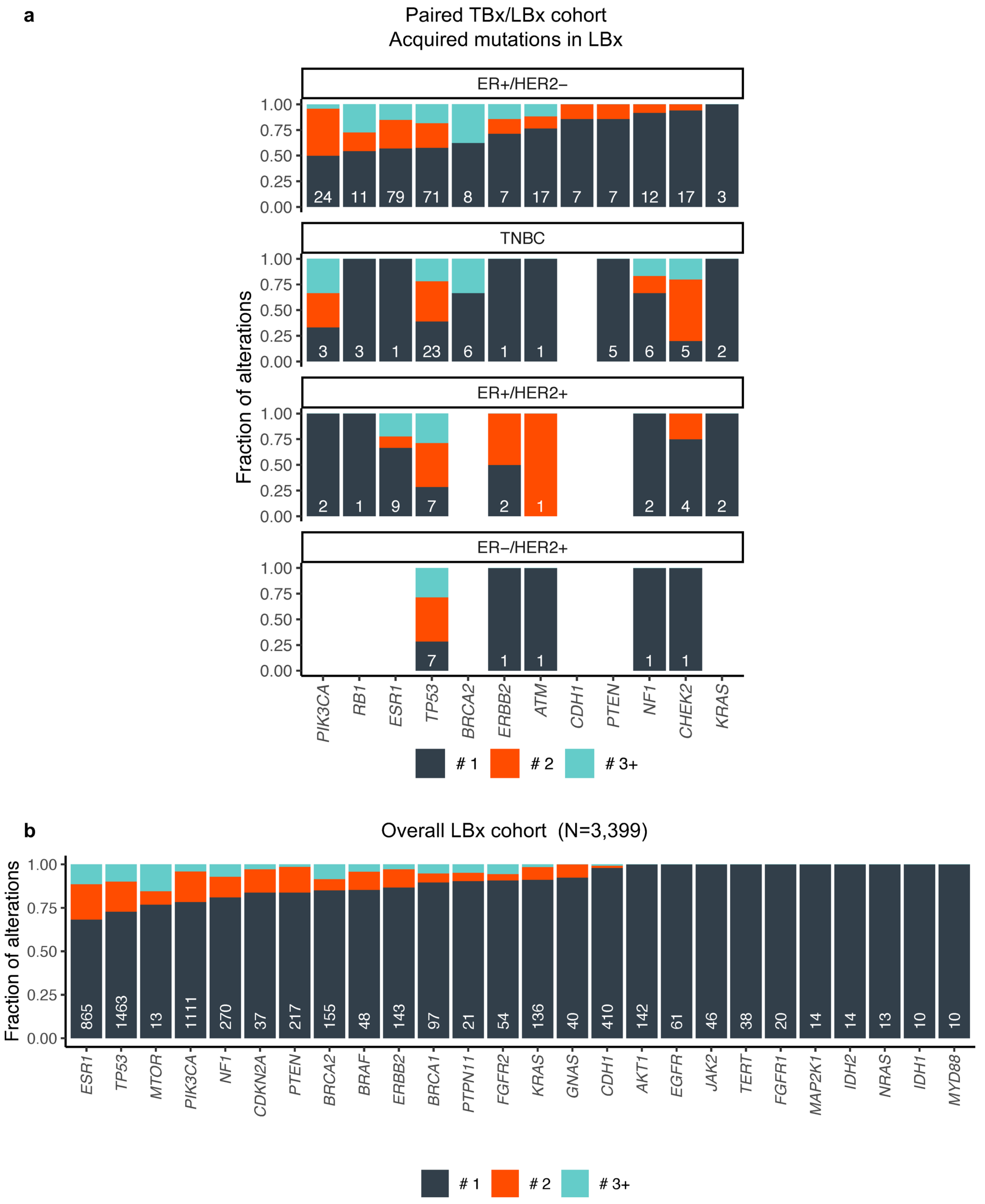

Supplementary Figure 12. Polyclonality of variants detected in liquid biopsies. (a) Stacked bar plots displaying the polyclonality of short variants acquired in follow-up liquid biopsies (LBx) in the paired biopsy cohort, based on receptor subtype status available for the prior tissue biopsy (TBx) sample, and **(b)** in the overall LBx cohort, based on the detection of one or more short variants within the same gene in a sample. The total number of samples is shown on each bar. Genes are ordered by the extent of polyclonality.

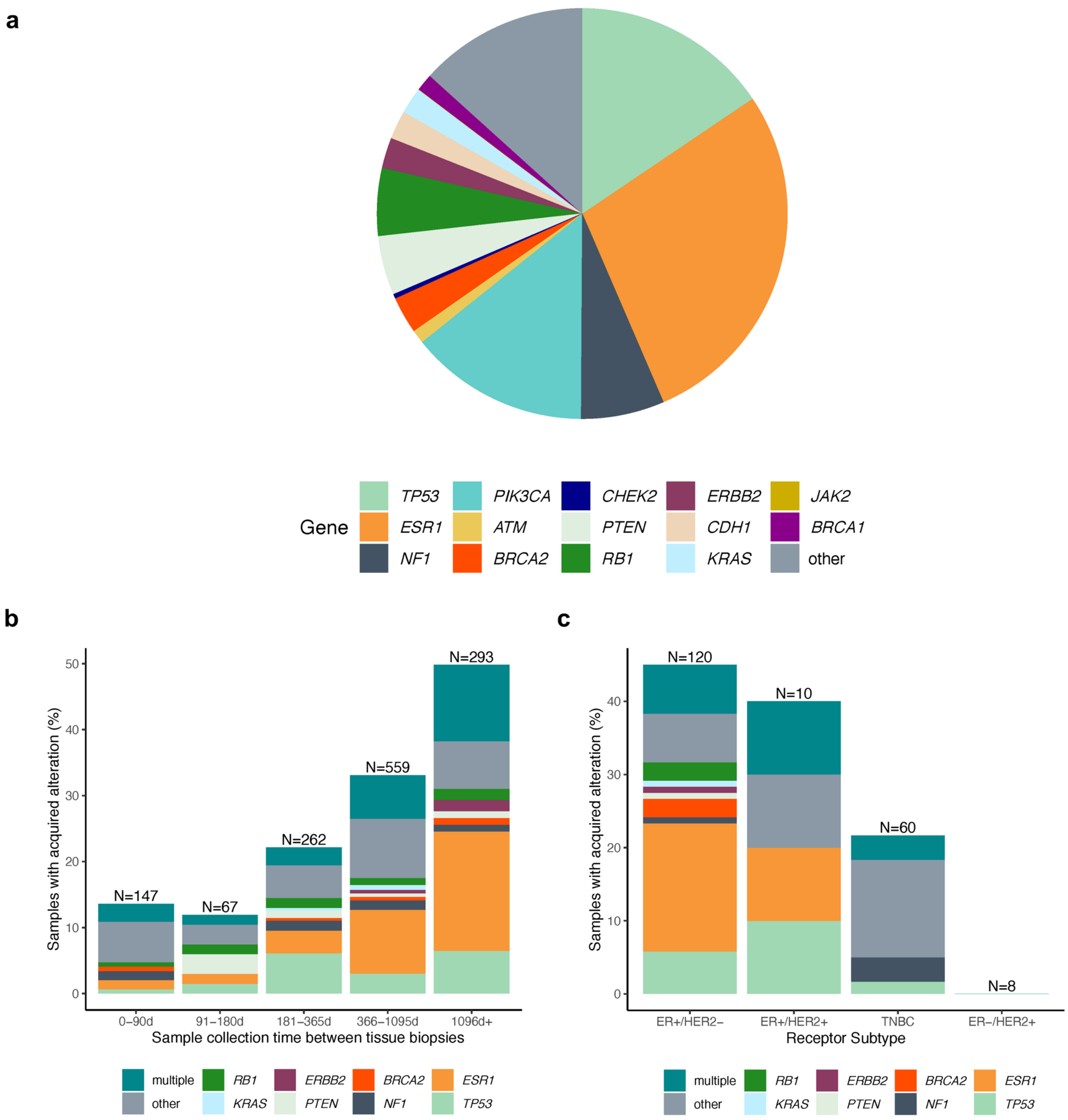

Supplementary Figure 13. Investigation of patient-matched tissue biopsies. (a) A pie chart displaying the spectrum of acquired short variants in 1,328 breast cancer patients with longitudinal tissue biopsies. Trends of acquired short variants based on **(b)** the time between biopsies, and **(c)** different receptor subtypes. The total number of samples in each analysis bin is displayed on top of each bar.

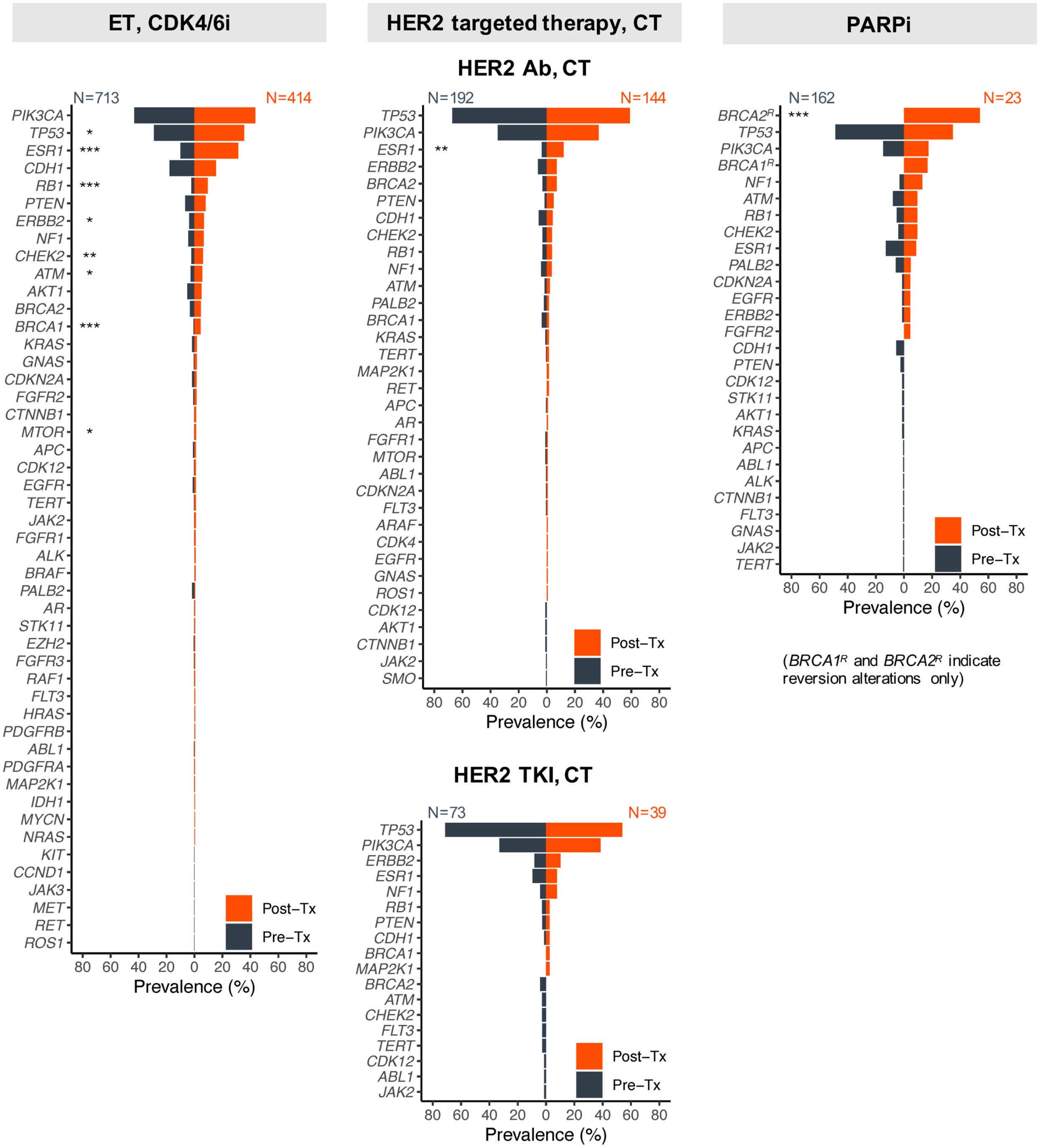

Supplementary Figure 14. Patterns of gene alterations pre- and post-treatment in the overall clinico-genomic database. Prevalence of gene short variants, comprising point mutations and short indels, for patient samples take pre- and post-treatment are shown in black and red bars respectively (*See Methods for additional details on cohort selection).* Only genes with at least one detected short variant are shown. The difference in prevalence of gene alterations for each treatment group, between pre- and post-therapy samples, was tested using a two-sided Fisher's exact test (p-value thresholds *: 0.05, **: 0.01, ***: 0.001). FDR adjusted p-values are provided in Supplementary Table S17 accompanying this analysis. The therapy groups include the following - endocrine therapy (ET) and Cyclin-dependent kinase inhibitors

(CDK4/6i), HER2 targeted therapy and chemotherapy (CT), Poly adenosine diphosphate-ribose polymerase inhibitors (PARPi). Patients receiving HER2 targeted therapy were further assessed based on the specific type of intervening therapy: antibody (Ab)-drug conjugates or tyrosine kinase inhibitors (TKI). Of note, only *BRCA1* and *BRCA2* reversions ($BRCA1^R$, $BRCA2^R$) are shown in the PARPi category.

REFERENCES

1. Woodhouse, R. *et al.* Clinical and analytical validation of FoundationOne Liquid CDx, a novel 324-Gene cfDNA-based comprehensive genomic profiling assay for cancers of solid tumor origin. *PLoS One* **15**, e0237802 (2020).
2. Antonarakis, E. S. *et al.* Prevalence of inferred clonal hematopoiesis (CH) detected on comprehensive genomic profiling (CGP) of solid tumor tissue or circulating tumor DNA (ctDNA). *J. Clin. Oncol.* **39**, 3009–3009 (2021).
