# Supplementary material for: Tissue and liquid biopsy profiling reveal convergent tumor evolution and therapy evasion in breast cancer
Source: Nat Commun. 2022 Dec 5;13:7495. doi: 10.1038/s41467-022-35245-x (PMC9723105; doi:10.1038/s41467-022-35245-x)
Supplement: Supplementary file 3 — Description of Additional Supplementary Files [file 41467_2022_35245_MOESM3_ESM.pdf]

Description of Additional Supplementary Files

Supplementary Data 1: Histo-pathological features of the overall tissue biopsy cohort

Supplementary Data 2: Commonly targeted genes for tissue and liquid biopsy assays

Supplementary Data 3: Prevalence of short variants in genes for samples profiled on each biopsy platform

Supplementary Data 4: Prevalence of short variants in genes for samples profiled on each biopsy platform across different receptor subtypes

Supplementary Data 5: Prevalence of different classes of gene alterations in tissue and liquid biopsies

Supplementary Data 6: Patterns of co-occurrence and mutual exclusivity between gene alterations for each biopsy platform

Supplementary Data 7: Prevalence of gene alterations in samples profiled on the recently approved FoundationOne®Liquid CDx assay

Supplementary Data 8: Characteristics of the patient-matched tissue and liquid biopsy cohort

Supplementary Data 9: Percent positive agreement (PPA) for liquid biopsies based on different factors

Supplementary Data 10: Patterns of acquired alterations in patient-matched liquid biopsies with prior tissue biopsy profiling

Supplementary Data 11: Patterns of frequently acquired gene alterations detected in liquid biopsies based on different factors

Supplementary Data 12: Patterns of *PIK3CA* short variants in patient-matched biopsies

Supplementary Data 13: Mechanisms of *BRCA1/2* reversions identified in the overall liquid biopsy cohort among cases with multiple *BRCA1/2* short variants

Supplementary Data 14: Examination of read support in the matched tissue biopsy for variants uniquely identified in the liquid biopsy sample

Supplementary Data 15: Patterns of gene alterations in patients with longitudinal tissue biopsies

Supplementary Data 16: Patterns of frequently acquired gene alterations based on different factors in patients with longitudinal tissue biopsies

Supplementary Data 17: Patterns of acquired gene alterations based on intervening therapy in the clinico-genomic database for patients with longitudinal biopsies

Supplementary Data 18: Patterns of gene alterations for any samples pre- and post-intervening therapy in the clinico-genomic database
