# Supplementary material for: Tissue and liquid biopsy profiling reveal convergent tumor evolution and therapy evasion in breast cancer
Source: Nat Commun. 2022 Dec 5;13:7495. doi: 10.1038/s41467-022-35245-x (PMC9723105; doi:10.1038/s41467-022-35245-x)
Supplement: Supplementary file 5 — Reporting Summary [file 41467_2022_35245_MOESM5_ESM.pdf]

nature portfolio

Corresponding author(s): Smruthy Sivakumar, Ethan Sokol

Last updated by author(s): Nov 21, 2022

Reporting Summary

Nature Portfolio wishes to improve the reproducibility of the work that we publish. This form provides structure for consistency and transparency in reporting. For further information on Nature Portfolio policies, see our Editorial Policies and the Editorial Policy Checklist.

Statistics

For all statistical analyses, confirm that the following items are present in the figure legend, table legend, main text, or Methods section.

n/a	Confirmed	
☐	☒	The exact sample size (n) for each experimental group/condition, given as a discrete number and unit of measurement
☐	☒	A statement on whether measurements were taken from distinct samples or whether the same sample was measured repeatedly
☐	☒	The statistical test(s) used AND whether they are one- or two-sided<br>*Only common tests should be described solely by name; describe more complex techniques in the Methods section.*
☒	☐	A description of all covariates tested
☐	☒	A description of any assumptions or corrections, such as tests of normality and adjustment for multiple comparisons
☐	☒	A full description of the statistical parameters including central tendency (e.g. means) or other basic estimates (e.g. regression coefficient) AND variation (e.g. standard deviation) or associated estimates of uncertainty (e.g. confidence intervals)
☐	☒	For null hypothesis testing, the test statistic (e.g. F, t, r) with confidence intervals, effect sizes, degrees of freedom and P value noted<br>*Give P values as exact values whenever suitable.*
☒	☐	For Bayesian analysis, information on the choice of priors and Markov chain Monte Carlo settings
☒	☐	For hierarchical and complex designs, identification of the appropriate level for tests and full reporting of outcomes
☒	☐	Estimates of effect sizes (e.g. Cohen's d, Pearson's r), indicating how they were calculated

Our web collection on statistics for biologists contains articles on many of the points above.

Software and code

Policy information about availability of computer code

Data collection	Data were collected by examining the Foundation Medicine database comprising patients who received comprehensive genomic profiling during routine clinical care. For a subset of patients, additional clinical data were collected by examining the Flatiron Health-Foundation Medicine Clinico-genomic database.
Data analysis	Statistics, computation, and plotting were carried out using Python 2.7 (Python Software Foundation) and R 3.6.1 (R Foundation for Statistical Computing).

For manuscripts utilizing custom algorithms or software that are central to the research but not yet described in published literature, software must be made available to editors and reviewers. We strongly encourage code deposition in a community repository (e.g. GitHub). See the Nature Portfolio guidelines for submitting code & software for further information.

Data

Policy information about availability of data

All manuscripts must include a data availability statement. This statement should provide the following information, where applicable:

- Accession codes, unique identifiers, or web links for publicly available datasets
- A description of any restrictions on data availability
- For clinical datasets or third party data, please ensure that the statement adheres to our policy

The sequencing data generated in this study is derived from clinical samples. The data supporting the findings of this study are provided within the paper and its supplementary files. All supplementary information accompanying the different analyses and figures presented in this study are provided in the Supplementary Data files. Due to HIPAA requirements, we are not consented to share individualized patient genomic data, which contains potentially identifying or sensitive patient information. Foundation Medicine is committed to collaborative data analysis, and we have well-established, and widely utilized mechanisms by which investigators

can query our core genomic database of >600,000 de-identified sequenced cancers to obtain aggregated datasets. Requests for collaborative datashares can be made by contacting the corresponding author(s) and filling out a study review committee form. Once approved, investigators are required to sign a data transfer agreement. Written proposals are considered at monthly meetings and data transfer agreements expire 18 months from execution of the agreement.

Field-specific reporting

Please select the one below that is the best fit for your research. If you are not sure, read the appropriate sections before making your selection.

☒ Life sciences ☐ Behavioural & social sciences ☐ Ecological, evolutionary & environmental sciences

For a reference copy of the document with all sections, see nature.com/documents/nr-reporting-summary-flat.pdf

Life sciences study design

All studies must disclose on these points even when the disclosure is negative.

Sample size	All available samples were included and no sample size calculation was performed. This study includes breast cancer samples analyzed by Foundation Medicine at the time of the study: 29,704 patients with tissue biopsy comprehensive genomic profiling (CGP), 3,339 patients with liquid biopsy CGP and 712 with longitudinal tissue and liquid biopsy CGP were examined. Treatment information was retrospectively reviewed in an independent de-identified clinico-genomic database comprising 7,681 patients with breast cancer.
Data exclusions	Samples with a failed clinical report were excluded.
Replication	Because the data are from a very large cohort of patient samples, the analyses cannot be replicated.
Randomization	This is an observational study, so no randomization was performed.
Blinding	No participants were allocated to groups.

Reporting for specific materials, systems and methods

We require information from authors about some types of materials, experimental systems and methods used in many studies. Here, indicate whether each material, system or method listed is relevant to your study. If you are not sure if a list item applies to your research, read the appropriate section before selecting a response.

Materials & experimental systems

n/a	Involved in the study	
☒	☐	Antibodies
☒	☐	Eukaryotic cell lines
☒	☐	Palaeontology and archaeology
☒	☐	Animals and other organisms
☐	☒	Human research participants
☒	☐	Clinical data
☒	☐	Dual use research of concern

Methods

n/a	Involved in the study	
☒	☐	ChIP-seq
☒	☐	Flow cytometry
☒	☐	MRI-based neuroimaging

Human research participants

Policy information about studies involving human research participants

Population characteristics	Detailed description of the cohort characteristics is outlined in Figure 2.
Recruitment	Patients with breast cancer who underwent testing as part of routine clinical care and consented for research were included in this study. Genomic profiling is typically performed later in critical care, so the data may be biased for later-stage cases. Additionally, longitudinal profiling dataset may be biased for patients with a longer survival to receive multiple tests.
Ethics oversight	Approval for this study, including a waiver of informed consent and Health Insurance Portability and Accountability Act waiver of authorization, was obtained from the Western Institutional Review Board (Protocol #20152817). The Institutional Review Board granted a waiver of informed consent under 45 CFR § 46.116 based on review and determination that this research meets the following requirements: (i) the research involves no more than minimal risk to the subjects; (ii) the research could not practicably be carried out without the requested waiver; (iii) the waiver will not adversely affect the rights and welfare of the subjects. For the clinico-genomic database (CGDB), IRB approval of the study protocol was obtained prior to study conduct and included a waiver of informed consent based on the observational, non-interventional nature of the study (WCG IRB, Protocol No. 420180044). No compensation was provided as samples were obtained during routine clinical care.

Note that full information on the approval of the study protocol must also be provided in the manuscript.
